# Organoid Cultures In Silico: Tools or Toys?

**DOI:** 10.3390/bioengineering10010050

**Published:** 2022-12-30

**Authors:** Torsten Thalheim, Gabriela Aust, Joerg Galle

**Affiliations:** 1 Interdisciplinary Institute for Bioinformatics (IZBI), Leipzig University, Härtelstr. 16–18, 04107 Leipzig, Germany; 2 Department of Surgery, Research Laboratories, Leipzig University, Liebigstraße 20, 04103 Leipzig, Germany

**Keywords:** organoids, computational multiscale models, stem cell organization, disease modelling, drug response

## Abstract

The implementation of stem-cell-based organoid culture more than ten years ago started a development that created new avenues for diagnostic analyses and regenerative medicine. In parallel, computational modelling groups realized the potential of this culture system to support their theoretical approaches to study tissues in silico. These groups developed computational organoid models (COMs) that enabled testing consistency between cell biological data and developing theories of tissue self-organization. The models supported a mechanistic understanding of organoid growth and maturation and helped linking cell mechanics and tissue shape in general. What comes next? Can we use COMs as tools to complement the equipment of our biological and medical research? While these models already support experimental design, can they also quantitatively predict tissue behavior? Here, we review the current state of the art of COMs and discuss perspectives for their application.

## 1. Introduction

Organoids are 3D self-organizing multicellular aggregates that differentiate in vitro into functional cell types, recapitulating the structure and function of the tissue of origin [1]. They can be established from embryonic (ESC), induced-pluripotent (iPSC) or adult stem cells (ASCs). While ESC- and iPSC-derived organoids involve stepwise differentiation protocols that resemble SC regulation during gastrulation and organogenesis, ASC-derived organoids require media supplemented with signal pathway activators and/or inhibitors that support SC maintenance under tissue homeostasis. Landmarks in the organoids field were ESC-derived cortical tissues [2] and organoids derived from ASCs of the small intestine [3]. Meanwhile, organoids exist for many other tissues including heart, liver, lung, mammary gland, pancreas, prostate, skin, stomach and more [4]. An organoid cell atlas has been envisioned [5]. In our review, we focus on organoids that resemble fetal, adult or tumor tissue. We do not include gastruloids that resemble tissues at early embryonic development [6] and tumor spheroids that emulate aspects of 3D tumor mass expansion only [7].

**Figure 1:**
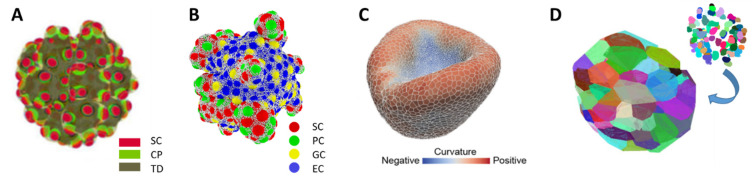
**Methods for modelling 3D cell aggregates.** (**A**) Continuum model of a colon cancer organoid [10]. Colors indicate the density of different cell types. SC: stem cell, CP: committed progenitor, TD: terminal differentiated cell. (**B**) Individual cell-based model of an intestinal organoid [11]. Cells are represented by overlapping spheres. Colors indicate different cell types. PC: Paneth cell, GC: goblet cell, EC: enterocyte. Cell motion is confined by a polymer network enveloping all cells (grey). (**C**) Vertex model of an optical-cup [12]. Cells are represented by prisms that form a dense surface. (**D**) 3D Voronoi tessellation of a tumor spheroid [13]. The cell shape is derived from the neighborhood relations of experimentally detected cell centers (upper right). Individual cells are indicated by different color.

In order to gain mechanistic insight into organoid formation and to support culture design computational organoid models (COMs), i.e., in silico counterparts of organoids, have been developed in the last decade [8,9]. The approaches are as diverse as the computational methods typically used to describe 3D cell aggregates (Figure 1). Differential equation-based continuum models describe cell aggregates based on spatio-temporal density distributions of cell types [10] (Figure 1A). In contrast, individual cell-based models represent each cell as a 3D object. This model class includes models where cells of defined shape can move, proliferate and differentiate into several lineages [11] (Figure 1B), but also vertex-based approaches where cells have very flexible shape enabling a better approach to the mechanics of dense tissue [12] (Figure 1C). In order to take cell composition of aggregates directly from experiments, Voronoi tessellation can be used to define cell shape based on the distribution of the cell nuclei [13] (Figure 1D). Notably, this method has been applied in 2D COMs only. Advantages and disadvantages of these methods are summarized in Table 1. COMs based on these approaches typically address questions about tissue self-organization. Frequently, they build on computational models of the tissue of origin [14,15,16,17]. COMs with simpler, often fixed geometry were introduced to inform experimentalist how to optimize oxygen and nutrient transport [18] or drug delivery [19].

While there is no doubt about the value of organoids in basic and applied cell biology, the question remains, what can we learn from their in silico counterparts?

## 2. COMs of Organoid Formation

From a basic research point of view, organoids are an exceptional model to study the interplay between SC organization and tissue shape, i.e., tissue self-organization. First COM studies focused on intestinal tissue. Organoids of this mono-layered epithelium were first established by T. Sato [3]. This study demonstrated that in vitro within Matrigel in vivo-like structures of crypts and villi can form, which contain all functional cell types including Paneth cells (PC), goblet cells (GC), enterocytes (ECs) and others. A challenge regarding modeling of these organoids is their changing size and shape during culture.

Asking for the modes of self-organization, Buske et al. [20]. developed the first 3D single-cell-based COM of an intestinal organoid. In contrast to their intestinal crypt model [17], in this COM the basal membrane is no longer represented by a rigid wall but by a polymer network that is capable of re-organization in response to forces originating in growth and movement of the cells attached to it. These dynamics enable organoid budding being induced by PCs, which differ from SCs and other cells by attaching more strongly to the polymer network and thereby inducing its softening and spontaneous bending. COM studies by Pin et al. [21] and Almet et al. [22] suggest a similar function of PCs, although their approaches differ. Pin et al. [21] assumes incompressible cells and different cell–cell contact-driven deformation behavior for SCs and PCs. If PCs are less deformable, cell growth leads to lateral SC protrusion out of the spherical cross-section of an existing crypt/organoid. Reaching a defined size, these protrusions are considered as irreversible, initial buds of a new crypt. The bud number increases for decreasing PC deformability. Almet et al. [22] assumes compressible cells and higher stiffness of PCs compared to SCs. For increasing cell numbers, the initially circular organoid cross-section changes its shape. Sufficient deviation from circular shape is considered as a fission event, the frequency of which increases for increasing differences in stiffness.

All these studies show that the specification of a mechanically unique cell type is essential for shape transitions. In particular, their findings suggest that differentiated PCs do not only support SC self-renewal by secreting growth factors [3], but in addition add a specific mechanical component determining the shape of the intestinal SC niche.

**Figure 2:**
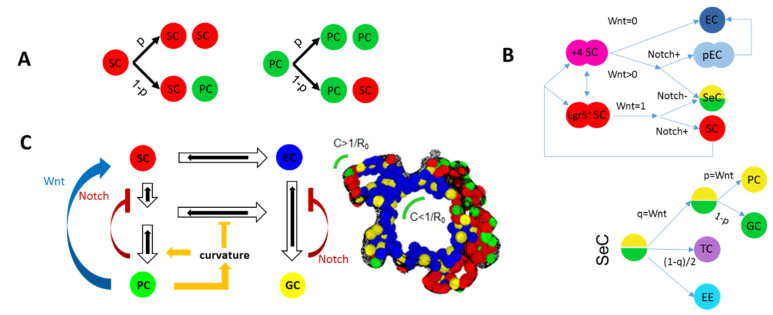
**SC specification and differentiation in intestinal COMs.** (**A**) Intrinsic regulation as suggested by Almet et al. [22]. Two types of cells (SC: stem cell (soft), PC: Paneth cell (hard)) divide asymmetric or symmetric with a defined probability p Є [0, 1]. (**B**,**C**) Environmentally regulated fate: (**B**) Detailed scheme provided by Pin et al. [16,21]. Dividing SCs self-maintain or irreversibly specify into absorptive or secretory cells/progenitors depending on externally provided Wnt and Notch signaling. Further specification into different secretory cells is controlled by the probabilities p and q that are Wnt-dependent. SeC: secretory cell, GC: goblet cell, TC: tuft cell, EE: enteroendocrine cell, pEC: enterocyte progenitor, EC: enterocyte. (**C**) Tissue shape-dependent regulation [20]. Fully reversible lineage specification is linked to Wnt and Notch signaling. PC specification in addition requires a threshold tissue curvature: C > 1/R_0_. R_0_: local curvature radius of the tissue.

As intestinal organoids grow from SCs, the intestinal COMs mentioned above consider SC specification and differentiation explicitly. However, the in silico approaches are again very diverse. Almet et al. [22] assume a simple intrinsic model of intestinal SCs. Here, maintenance of soft SCs and specification of hard PCs occurs following cell division with fixed probabilities. In contrast, Pin et al. [16,21] and Buske et al. [20]. suggest a completely extrinsic regulation depending on the activity of the Wnt and Notch pathway (Figure 2). Their models differ in the assumptions regarding time point and reversibility of the decisions and the point that Buske et al. [20] introduced a tissue curvature-dependent regulation of PC specification. Regardless of these differences, the cell composition of all models self-organizes via neighborhood properties and their parameters can be adjusted to obtain a realistic PC and SC distribution being sufficient to obtain shape transitions as budding.

In summary, basic features of intestinal organoids can be recapitulated using very different approaches. Shape changes can be induced by changing parameters of the biomechanical or the lineage specification model. Although the models by Buske et al. [20] and Pin et al. [16,21] integrate activity of the Wnt and Notch pathway, they neglect their crosstalk [23]. Thus, none of the models allows for quantitative modeling of state perturbations by pathway regulation.

**Figure 3:**
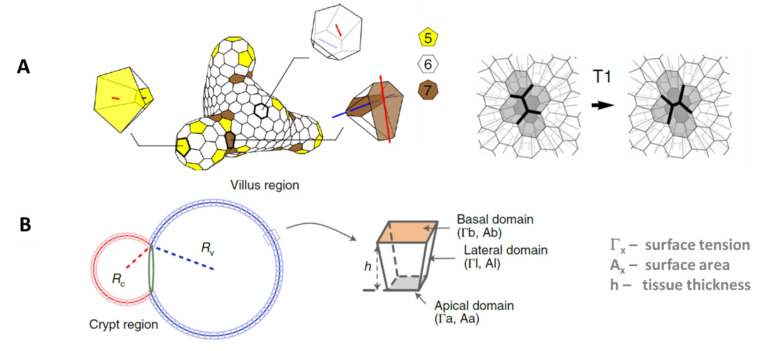
**Vertex models of organoid-like cell aggregates.** (**A**) Organoids in the model by Rozman et al. [27] have a flexible shape. The cells can have different numbers of neighbors. Examples of 5-, 6- and 7-coordinated cells are magnified. Interconversions can occur via so-called active T1 transitions that change the connectivity of the apical network (top view), followed by an update of the basal network and the lateral sides. (**B**) Schematic of the organoid model introduced by Yang et al. [31]. Two hollow spheres in contact represent the crypt (radius R_c_) and villus region (radius R_V_). Each cell has four neighbors. Due to the positive curvature, the apical surface of each cell, which is directed towards the lumen, is smaller than its basal surface. Figure 3B is published under Creative Commons CC-BY-NC-ND, all rights are reserved by the publisher.

Subsequent to intestinal COMs, COMs of other tissues have been introduced including optic-cup [12], lung [24] and pancreas [25]. The COMs by Sachs et al. [24] and Hof et al. [25] aimed at explaining selected dynamic phenomena of organoid culture. Sachs et al. [24] simulated planar cells moving on the surface of a sphere and linked experimentally observed active organoid rotation to correlated motion of these cells. Hof et al. [25] introduced mechanically cross-linked cells on a spherical shell and explained volume oscillations occurring during organoid growth by the interplay between their proliferation activity and the osmotic swelling of the shell. The approach to optic-cup development by Okuda et al. [12] addressed for the first time organoid self-organization for tissue other than intestinal tissue. In their study, Okuda et al. [12] identified mechanical feedback as an essential component of robust optic-cup organoid morphogenesis as well. In contrast to the early intestinal COMs, they described the organoid cells using a vertex model [26]. This model type became the favorite COM type in the following years. In a more general study, Rozman et al. [27] used this model type to identify the conditions at which organoid shape transitions, such as budding, occur. They found that spontaneous budding requires frequent active reorganization of lateral cell–cell interactions leading to so-called ‘tissue fluidization’. During budding, they observed local enrichment of vertex-types (Figure 3A). Although not addressed by Rozman et al. [27] themselves, these different cell–cell contact structures might at least in part trigger cell differentiation [28]. Therewith, the model provides an alternative to the assumption of curvature dependent PC differentiation by Buske et al. [20] (Figure 2C).

Recent experimental results validate and further detail the role of SC specification and differentiation for spatio-temporal organization of organoids. In intestinal organoids, the SC niche pushes the extracellular matrix and folds through apical constriction, whereas the transient amplifying zone pulls the extracellular matrix and elongates through basal constriction [29]. The budding process of the organoid does not start before an inflation collapse occurs [30]. In a combined experimental-theoretical study, Yang et al. [31] demonstrated that even simplified vertex models are capable of integrating this kind of data and can predict tissue shape based on them. Testing different model assumptions, they predict that spontaneous curvature is the major driving force of crypt formation. However, in their model, crypt and villus-like regions are both spherical in general (Figure 3B). Thus, they are not well suited to study feedback between mechanics and lineage specification.

The mentioned COM studies shed light on how mechanical and osmotic forces results in specific organoid shape and demonstrated that this shape depends on cell composition. By testing alternative hypotheses, their simulation provided a mechanistic understanding of different aspects of organoid maturation. Similar approaches might be applied to explain stable morphological heterogeneity of organoid culture as observed for mammary gland organoids [32] or to study morphological differences of organoids derived from closely related tissues such as different salivary glands [33]. In any case, a strong feedback of cell mechanics and metabolism on fate decisions can be expected, which currently remains largely unexplored. Thus, the question remains whether state of the art COMs can support clinical applications of organoids.

## 3. Optimizing Organoid Culture

Clinical applications of organoids require, first of all, their massive expansion. Therefore, a major challenge is organoid culture optimization, e.g., to avoid insufficient nutrient or oxygen supply. Modelling approaches with that aim have a long tradition (single-cell-based models [34], continuum models [35]). Recently, such modelling has been undertaken to optimize organoid culture [36]. Introducing a continuum model of a bioreactor, Ellis et al. [36] modelled the spatial temporal distribution of the key metabolites glucose and lactose by calculating consumption, advection and diffusion of them (reaction-advection-diffusion model). Systematically simplifying their model, they identified dominant mass transport mechanisms and opened an efficient way to inform bioreactor operating conditions for different organoid types. In their model, organoid cells have defined growth and metabolite consumption rates. The authors envision culture models that take more detail into account, e.g., time-dependent rates.

While these studies aimed at optimizing the environment, other studies focused on a better understanding of nutrient or drug transport inside organoids. Actually, organoid size is controlled, in addition to proliferation activity and lumen pressure (see above), by nutrient diffusion into the organoid and the accordingly achieved metabolism [18]. In cerebral organoids, these phenomena affect organoid self-organization being responsible, e.g., for spatial cell density variation. While such studies typically assume a simple shell structure of organoids [37], Leedale et al. [19], interested in optimized drug dosing, performed a 2D Voronoi tessellation of a cross-section of a liver organoid to involve real cell shape and packing. One may expect that transport problems are relevant for spheroids only and not for hollow spheres as intestinal organoids. However, there is some evidence that also growth of hollow organoids is affected by reduced oxygen supply [38]. The question arises to what extent such effects are considered by individual cell-based COMs.

**Figure 4:**
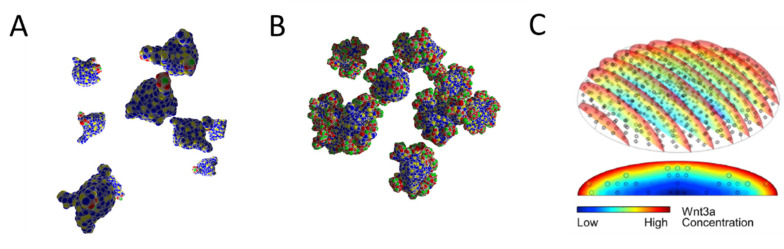
**Simulated populations of organoids.** The model provided by Thalheim et al. [11] enables controlling Wnt and Notch activity in the cells via cell–cell interaction. PCs provide both Wnt and Notch ligands which are necessary for SC maintenance. The number of contacts N required to stabilize the SC fate controls the SC fraction and thus organoid shape and growth velocity. (**A**) N = 1 after 42 days of culture, (**B**) N = 2 after 21 days of culture. These differences can be motivated by externally provided growth factors. Colors of the cells as in Figure 1B and Figure 2C. (**C**) In contrast to the assumptions by Thalheim et al. [11], the growth factors (here Wnt3a) are not homogeneously distributed in the culture [40], potentially resulting in mixtures of the growth types. Small circles: organoids. Figure 4C is published under Creative Commons CC-BY-NC-ND, all rights are reserved by the publisher.

The intestinal COMs detailed above consider growth factors to affect tissue organization. This assumption is based on experimental data on growth factors controlling SC lineage specification [39]. The models profit from the observation that tissue self-organization in organoids does not require growth factor gradients (e.g., Wnt gradients). Accordingly, they assume fixed growth factor concentrations throughout the culture and neglect properties that might depend on diffusion or other transport phenomena. An example can be found in studies on Wnt and Notch regulation in intestinal organoids [11]. The authors extended the model by Buske et al. [20] by considering effects of culture supplements such as R-spondin on Wnt activity (Figure 4A). However, while they assume constant Wnt activity in all organoids, recent experimental data demonstrate that a heterogeneous growth factor distribution arises even in a standard culture dish [40] (Figure 4B). Such heterogeneity might even be more pronounced during scaffold-guided organoid growth that has already been in practice for years [41]. Thus, future COMs will have to consider culture system-specific nutrient and growth factor distribution as well as organoid-specific metabolism to explain organoid growth.

These properties depend on the polymer matrix embedding the organoids. Existing COMs describe this matrix, e.g., by re-organizable polymer nets [11,20] or interconnected layers of non-epithelial cells [22]. While these approaches allow simulating mechanical properties of the matrix, they do not consider signaling mediated via cell linkage to specific matrix components [42]. Approaches that enable integrating this kind of signaling have been introduced [43]. However, the computational effort associated with such extensions is huge.

## 4. Steps towards In Silico Disease and Therapy Modelling

From a clinical application point of view, organoids are excellent disease models and can be used to study cell response to pathogens or drugs [44]. Currently, organoids are applied in studies regarding infectious diseases, genetic diseases and cancer. In particular, cancer studies benefit from analyses of tumor-derived organoids enabling patient specific drug testing and therapy decisions [45]. Here, COMs are considered an effective tool for bridging the preclinical to clinical translational gaps [46].

An obvious benefit of COMs in clinical COM applications arises from calculations of concentrations profiles of metabolites, nutrients and growth factors as discussed above, i.e., from pharmacokinetic modeling. Further benefits are methodical ones: Most COMs include at least in part stochastic decisions, e.g., regarding cell growth, division and lineage specification. Thus, simulated organoids vary in shape, size and cell type composition, i.e., display heterogeneity. Quantification of this heterogeneity can help to distinguish natural fluctuation-based heterogeneity from shape changes related to transcriptional deregulation. In recent years, high throughput platforms for organoid screening have been established together with methods for morphological classification of organoids based on machine learning such as MOrgAna [47] and Phindr3D [48]. Applying them to both in vitro and in silico culture could enable such comparative morphological studies. A prerequisite for these efforts is appropriate 3D representation of in silico organoids.

Shape heterogeneity of organoids originating in variation of intrinsic parameters has been analyzed in simulation studies of tumor organoids [49]. Such simulations might help to characterize the consequences of abnormal SC regulation and may support medical study design by defining the sample size required for pursued classifications.

In principle, COMs can also directly contribute to disease and therapy modeling, i.e., they can address questions of pharmacodynamics. Currently, however, there are many gaps to fill. Until now, computational modelling was not capable of keeping up with the rapid experimental advances in the organoid field. Even state of the art COMs have problems contributing to disease modeling. A major reason might be that multiscale tissue modelling still suffers from a shortage of methods that effectively break down molecular complexity and enable a straightforward link between molecular regulation and cellular function. Providing a (tissue specific) regulatory model of lineage specification suitable to become part of a computational model is thus still a field of research of its own. Thus, although most of the COMs base on a single cell-approach and accordingly are suitable for integrating, e.g., single cell RNA sequencing data [50,51], they only integrate very simple molecular regulation models (see above). Accordingly, their outcome often remains very general. We envision two essential steps towards future applications of COMs. First, the establishment of models that integrate aspects of metabolic and biomechanical regulation of SCs. Second, the development of sophisticated regulatory models of pathological cell function based on (single cell) Omics-data.

**Step 1**: An essential step towards COM-based disease modelling involves improved lineage specification models. The simplest way of integrating molecular details into COMs is to focus on single genes (master regulators) or pathways, and to link changes in their transcription/activity to decisions regarding lineage specification (as in the intestinal COMs discussed above). Studies on differentiation of cell populations demonstrated that robust decisions can thereby result from different feedback mechanisms, e.g., via cell cycle regulation [52] or epigenetic regulation [53]. Feedback of secreted growth factors on lineage specification within organoids has been investigated by use of a reaction-advection-diffusion approach [10].

A current challenge is to identify core regulatory circuits that link the essential lineage specification pathways [54]. Supervised and unsupervised methods that enable their identification based on omics-data are well established [55]. The complex regulatory behavior of such circuits, which may also include epigenetic regulation, can be simplified subsequently, e.g., by mapping it on a Boolean network, where time and space are discrete, as demonstrated for the regulation of iPSCs [56,57].

Recent experimental studies suggest that such circuits should also cover the feedback from organoid shape and matrix stiffness on lineage specification. So, Sen et al. [58] demonstrated feedback of early geometric confinement on lineage specification in cerebral organoids based on gene expression analysis. Among the pathways affected are Wnt and Notch, known master regulators of SC specification [54]. A study by Hushka et al. [59] showed that matrix softening supports budding and thus maturation of intestinal organoids. Here, the question remains whether soft matrix supports PC specification or facilitates high local curvature. The COM by Thalheim et al. [11] assumes PC-associated matrix softening to achieve robust budding. Alternatively, PCs might be specified preferentially in soft Matrigel regions. Additional studies on the role of mechanosensitive pathways for organoid formation are required to validate related assumptions.

Metabolism of SCs and their differentiated progeny is not only different; the differences can synergize to support robust SC self-renewal within organoids, as shown for intestinal SCs and PCs [60]. De novo fatty acid synthesis sustains intestinal SC function [61]. Thus, SC regulation circuits should also consider metabolic activity. Potential model strategies are established to integrate this kind of regulation into organoid models [62].

**Step 2:** Assuming defined regulatory states, i.e., defined cell types into which SCs specify, all quantitative differences of organoid behavior originate in changes of the fractions of them. A potential therapy would aim at restoring the changes in cell composition by drugs [63]. However, this approach covers SC specification-associated disease (including tumors) only. It does not include disease associated with loss or gain of function, in particular in differentiated cells. Clearly, if these changes do not provide strong feedback on lineage specification, basic analysis of related disease types does not require applying a COM. An example: Recently, a logical network-based drug-screening platform for Alzheimer disease has been developed to classify pathological features of human brain organoids [64]. A gene regulatory model was developed and treatment was simulated, i.e., drug response was studied, without considering organoid self-organization. In this example, reduced complexity of the regulatory circuit was achieved by focusing on major signaling pathways extracted from literature, which are associated with this disease. Alternatively, one might use machine learning for model establishment. However, in such an approach one would reduce mechanistic insight into the underlying regulation [55]. In general, integrating models of perturbed non-SC function into COMs might be a second order step only.

## 5. Summary

**Figure 5:**
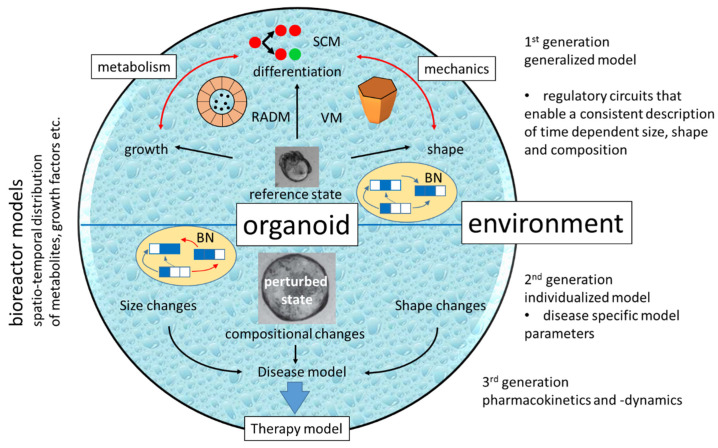
**In silico organoids.** Currently available are 1st generation COMs for different tissue. However, they often focus on a single aspect such as organoid differentiation, shape formation and limited growth. Accordingly, they use different approaches such as individual cell-based SC models (SCM), vertex models (VM) or reaction-advection-diffusion model (RADM). Missing are COMs combining these aspects in a core regulatory circuit, e.g., a Boolean network (BN), with a limited number of regulatory states. In 2nd generation COMs, individual model parameters and/or regulatory links are changed to simulate a disease model. Its behavior under therapy can then be tested combining it with a ‘bioreactor model’ describing the organoid’s environment.

Current COMs explain specific phenomena of organoid growth, shape formation or cell composition (Figure 5). In order to simulate organoid-based disease models, it might not be necessary to consider all these aspects. However, including different levels of SC regulation into COMs seems to be essential. Their specific requirements mutually confine the space of solutions of the model and can eventually result in a few stable states [53]. Screening the COM’s parameter space will then inform about the phenomena, which can be covered by the model and for which parameters they occur. In case these parameters depend on the environment, one can start a drug response simulation. The above considerations argue for COMs that combine multiscale feedback-models of SC differentiation (e.g., including transcriptional regulation by epigenetics and metabolic regulation of epigenetics) with models of differentiated cells, which describe their lineage specific metabolism and mechanics. Thereby, individual cells are probably best represented by vertex models because they straightforwardly enable considering feedback of tissue mechanics on lineage specification and allow inclusion of collective motion [26]. Such a COM needs to be further combined with reaction-advection-diffusion models to calculate local concentrations of nutrients and metabolites.

An obstacle to this approach might be a problem inherent to computational tissue modelling. The strength of a model approach is that it can provide simple hypotheses on tissue organization that are verifiable by experiments. For this purpose, models often use a level of abstraction that omits major variation. However, this kind of ‘noise’, i.e., these small variations are what most clinical applications are based upon. Here, the question arises whether COMs should reach an ever-increasing level of complexity to describe them. Beside the risk of implementation faults that might remain hidden, while increasing their complexity, the COMs would lose their most important feature of providing direct insight into tissue self-organization. A one-to-one representation of organoids in silico thus does not seem to be desirable. Approaches using methods of artificial intelligence might be better suited for such mapping. However, because transcription circuits can evolve in ways that preserve their output [65], complex states are not necessarily uniquely defined, resulting in limitations of such extensions as well.

A current trend in the application of organoids includes immune cell co-culture and microbiome studies [66]. Related COMs can be expected in the near future. They will need to consider the interaction of organoid cells with other cell types or pathogens. Whether these additional agents should be modeled individually is not clear.

Problems not addressed in COMs so far are: (i) mechanism of in vitro cell adaptation, (ii) changes associated with long-term organoid culture and (iii) effects of culture derived from aged individuals. A trend for enhanced in vitro compared to in vivo transformation has been shown not only for organoids originating from mice suffering from mismatch repair deficiency, but also for organoids derived from normal mice, although at a lower level [63]. Moreover, long-term epigenetic drifts are found in organoid culture, seemingly changing the competence to adapt to stress [67]. Thus, in silico modeling of organoid safety aspects might be a further challenge in the field.

In this review, we have not discussed technical questions of in silico modelling of organoids, as their implementation and computational power. Agent-based software frameworks for COMs have been discussed by Montes-Olivas et al. [8]. Clearly, according to the different levels of model complexity, the simulation effort in central processing unit (CPU-) time very much varies between the model classes. For complex individual cell-based models, it might reach the limits of currently available resources, limiting their broad application. Cell simulation techniques dedicated to running on graphics processing units might help to solve these problems [68].

## 6. Conclusions

In the past decade, computational modelling of tissue organization has very much benefited from organoid research. The new quality of data available on regulatory processes has enabled scientists to validate hypotheses on fundamental principles of tissue growth and self-organization and thus to improve in silico tissue modelling. While these studies extend our knowledge on cell metabolism, cell biomechanics and SC organization, the links between these aspects of tissue organization are still largely unknown. However, this knowledge is essential to understand self-organization of organoids, which is a prerequisite for simulation of pharmacological interventions and other perturbations of normal tissue function. Accordingly, support of clinical applications of organoids by COM approaches is mostly limited to pharmacokinetics. Thus, whether COMs will become essential tools in organoid based disease and therapy modeling remains to be seen in future. Currently, individual cell-based COMs are more or less nice toys for impactful visualization of related research questions.

## Figures and Tables

**Table 1:** Different 3D COM approaches (compare also Norfleet et al. [9]).

Organoid Described by:	Example	Pros	Cons
Spatio-temporal distribution of cell densities	Figure 1A	fast computation	Properties of individual cells cannot be addressed
Objects: spherical cells, polymer network	Figure 1B	simple interaction scheme	tissue mechanics hard to assess
Objects: prism-like cells	Figure 1C	well-defined tissue mechanics	limited cell mobility
Voronoi-polyhedrons generated from cell center distributions	Figure 1D	flexible cell shape direct from experiments	cell–cell interaction by center-center interaction

## Data Availability

Not applicable.
